# Association between non-high-density lipoprotein cholesterol to high-density lipoprotein cholesterol ratio (NHHR) with depressive symptoms: recent findings from NHANES 2005–2018

**DOI:** 10.3389/fpsyt.2024.1467142

**Published:** 2024-11-05

**Authors:** Chunyu Yan, He Wang, Changxing Liu, Jiamei Fu, Yabin Zhou

**Affiliations:** ^1^ First Clinical Medical College, Heilongjiang University of Traditional Chinese Medicine, Harbin, China; ^2^ Department of Cardiology, The First Hospital Affiliated to Heilongjiang University of Traditional Chinese Medicine, Harbin, China

**Keywords:** NHHR, depression, NHANES, US adults, cross-sectional study

## Abstract

**Background:**

The ratio of non-high-density lipoprotein cholesterol to high-density lipoprotein cholesterol (NHHR) index is a relatively new composite lipid index, the relationship between NHHR and depression is unclear from the current study. The primary aim of our study was to examine the association between the prevalence of depression and NHHR in a US population.

**Methods:**

The National Health and Nutrition Examination Survey (NHANES) provided the data for our investigation from 2005 to 2018. and primarily included participants who contained complete data on NHHR and depression in U.S. adults (age ≥20 years). Associations between NHHR and depression were assessed using multifactorial logistic regression analysis, subgroup analysis, and smoothed curve fitting.

**Results:**

In our study, 29,561 subjects in total showed a mean NHHR index of 3.12± 1.58,A noteworthy positive correlation was observed between NHHR and depression in multifactorial logistic regression analysis. Subgroup analyses and tests of interaction showed that gender, age, ethnicity, PIR, smoking, alcohol consumption, coronary heart disease, diabetes mellitus, hypertension, and stroke did not influence the NHHR and the association between depression (P for interaction > 0.05), whereas two stratification factors, BMI and sleep disturbance, may be potential factors in the association between NHHR and depression (P for interaction < 0.05).

**Conclusion:**

According to our present study, if the level of NHHR rises in American adults, their likelihood of developing depression also increases.

## 1 Introduction

With over 35 billion people affected globally, depression has emerged as a major public health concern. It is also one of the main causes of disability and low quality of life (1–3).

It’s worth noting that the frequency of adult depression in the U.S. is increasing each year, affecting about 8% of adults in the U.S., and is higher in women than in men (4). A high incidence, a high clinical cure rate but a low treatment acceptance rate and a high relapse rate are the hallmarks of depression as a mental illness. Along with cognitive abnormalities that could interfere with day-to-day learning and work, the patient may also experience hallucinations and, in extreme circumstances, suicidal thoughts (5, 6). Depressed moods may cause immune, endocrine and neurological dysfunction in the body, which can increase susceptibility to disease and lead to death (6). According to a recent meta-analysis, depression raised the probability of dying by 50% (7). In addition, depression not only reduces work efficiency and productivity, but also costs the global economy $1 trillion each year (8). Studies in the United States have found that the economic burden of depression is 1.6 percent of gross domestic product. However, current treatment regimens remain inadequate for some patients, and some may experience intolerable side effects, making the discovery of new depression-related bioindicators crucial for the prevention and treatment of depression. Many studies have shown that the pathogenesis of depression is closely associated with inflammation, neuroimmune dysfunction, and abnormalities in lipid metabolism (9–12). Previous findings on the association of biomarkers in lipid metabolism with depression have however been controversial, with some studies reporting that Triglycerides (TG) have a positive correlation with the likelihood of developing depression, while low-density lipoproteins (LDL-C), total cholesterol (TC), and high-density lipoproteins (HDL-C) have a negative correlation (13–16), In contrast, Raised HDL-C levels were substantially positively correlated with depression in both sexes, according to a cross-sectional study (17). Jane E. Persons et al. concluded from a meta-analysis that there was a U-shaped relationship between LDL and depression when LDL was used as a continuous variable (18). Non-HDL-C mainly contains cholesterol from all atherogenic lipoprotein particles, apolipoproteins, very low-density lipoprotein cholesterol (VLDL-C), and IDL-C, and relevant studies have demonstrated that the major components contained within non-HDL-C are linked to an increased risk of suffering from depression (19).

Therefore, the development of newer and more reliable lipid parameters is necessary for the prediction and management strategies of depression. The ratio of non-HDL-C to HDL-C (NHHR) is a newly discovered lipoprotein ratio that is known to assess atherosclerosis and blood predictors of cardiovascular disease risk (20). To date, Reports evaluating the connections between NHHR and depression are nonexistent.

Our investigation’s primary goal was to examine the relationship between NHHR and the prevalence of depression in participants from 2005-2018 using data from the National Health and Nutrition Examination Survey (NHANES). We postulated that elevated NHHR is connected to increased prevalence of depression.

## 2 Materials and methods

### 2.1 Study population

The National Center for Health Statistics’ main program, NHANES, or the National Health and Nutrition Examination Survey, is intended to evaluate the health and nutritional status of adults and children in the country. The survey offers comprehensive biological, psychological, behavioral, and demographic data at no cost by combining home interviews with health examinations. Written informed consent was acquired by each participant in this investigation, and the National Center for Health Statistics (NCHS) Ethics Review Committee approved the study procedure. For detailed data on participants, see http://www.cdc.gov/nchs/nhanes/index.htm.

**Figure 1:**
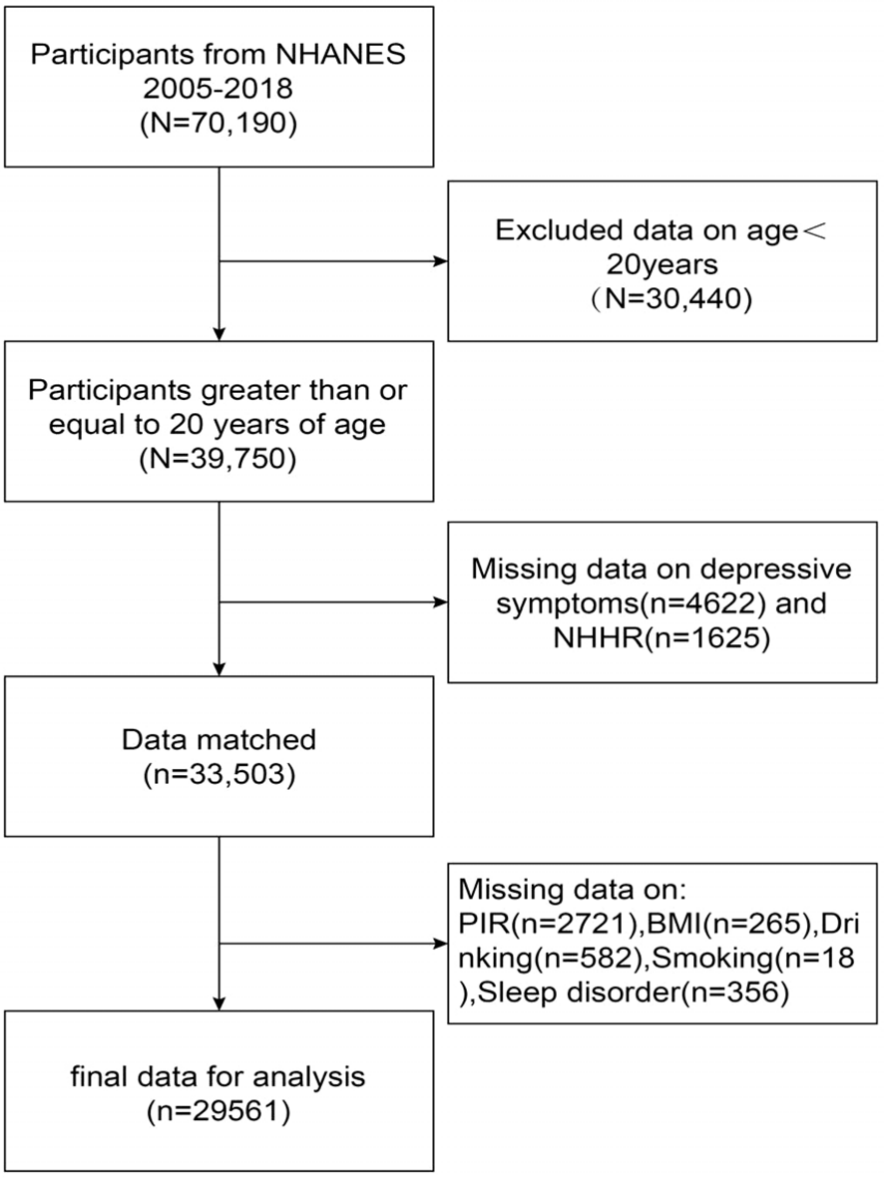
NHANES participant selection flowchart 2005-2018.

Our study used NHANES data from 2005 to 2018 and initially included 70,190 participants who received thorough data on NHHR and depression, after excluding individuals under the age of twenty (n=30440), participants missing information on the Depression Health Questionnaire-9 (PHQ-9) (n=4622) (n=4622) and NHHR (n=1625), and participants missing information on relevant covariates (PIR, BMI, smoking, drinking, and sleep disorders) (n=3942), resulting in a total of 29,561 participants who met the inclusion criteria, as shown in **Figure 1**.

### 2.2 Assessment of depressive symptoms

This study assessed depressive severity based on participants’ health status over the past two weeks through the Depression Screening Scale (PHQ-9).The PHQ-9 contains nine items, each scored 0-3, and the total score is the sum of the nine items added together. Participants with a score of 10 or more were diagnosed with depressive symptoms (21).

### 2.3 Assessment of NHHR

NHHR was used as the exposure variable in this study and was calculated by subtracting the HDL level from the total cholesterol level and dividing the resulting NHHR level by the HDL level to obtain the final NHHR data for the participants.

### 2.4 Treatment of covariates

In our current study, there were a number of potential covariates that could have been confounders of the association between NHHR and depression. Covariates included age (years), gender (male/female), race, education level, poverty income ratio (PIR), marital status, body mass index (BMI, kg/m^2^), smoking status (based on having smoked at least 100 cigarettes in a lifetime, participants who answered yes were considered smokers), drinking status (based on having had at least 12 alcoholic beverages in a lifetime, participants who answered yes were considered drinkers), total cholesterol (TC), sleep disorders, hypertension, diabetes, coronary heart disease, and stroke. Detailed data on all of the above latent variables are publicly available at www.cdc.gov/nchs/nhanes/.

### 2.5 Statistical analyses

The CDC’s criteria were followed in performing all statistical analyses, which included applying proper NHANES sampling weights and taking into account a complex multistage whole cohort survey design (22).

Continuous variables were expressed as mean ± standard deviation (SD) and categorical variables as percentages, and differences between the two control groups by whether the participant had depression or not were assessed using a weighted Student-t test (for continuous variables) or a weighted chi-square test (for categorical data). Multifactor logistic regression models were used to analyze the independent relationship between NHHR and the prevalence of depression. Model 0 was unadjusted for variables, model 1 was adjusted for gender, age, and ethnicity, and model 2 adjusted for marriage, education, alcohol and smoking status, PIR, BMI, total cholesterol, sleep disorders, hypertension, diabetes mellitus, coronary heart disease, and stroke in addition to model 1. Smoothed curve fitting was used to further analyze the linear relationship between NHHR and the prevalence of depression. Subgroup analyses stratified by age, sex, race, smoking status, drinking status, PIR, BMI, sleep disorders, hypertension, coronary heart disease, diabetes mellitus, and stroke were also performed by stratified multivariate regression analyses, and we added an interaction test to test for heterogeneity in the associations between subgroups, with P < 0.05 considered statistically significant. In addition, we used smooth curve fitting to predict whether there was a positive association between NHHR and depression prevalence stratified by BMI and sleep disturbance. All statistical data in this study were analyzed and visualized using R4.3.2 software for (http://www.R-project.org) and Empower Stats (http://www.empowerstats.com), Empower Stats uses gam () function in mgcv package to draw smooth curve fitting, and the curve fitting items are defined by s () function. and statistical significance was set at P < 0.05.

## 3 Results

### 3.1 Baseline characteristics of participants

**Table 1** depicts the weighted demographic baseline characteristics of the included participants. A total of 29,561 subjects were included in our study, of whom 49.09% were male and 50.91% were female, with a mean age of 49.45 ± 17.66 years. We set depression as a dichotomous variable, and compared to the non-depressed group, depressed subjects were characterized by a predominance of females, lower age, more often seen in other Hispanic and non-Hispanic blacks, more education in grades 9-12 and high school graduation/GED or equivalent, lower household income, widowed/divorced/separated/never married/living with a partner, higher rates of smoking and alcohol use, increased BMI, higher TC levels, and prevalence of sleep disorders, hypertension, coronary heart disease, diabetes, and stroke, with statistically significant differences in variables between groups (p < 0.05).

**Table 1:** Clinical characteristics of depressed and non-depressed patients (n=29561): NHANES, 2005–2018.

Characteristic	Total (N=29561)	Non- depressive symptoms (N=26990)	Depressive symptoms (N=2571)	*P*-value
Gender(%)				<0.001
Male	14513(49.09)	13587 (50.34)	926 (36.02)	
Female	15048(50.91)	13403 (49.66)	1645(63.98)	
Age (years), Mean (SD)	49.45 ± 17.66	49.41 ± 17.83	48.66 ± 16.04	0.042
Race (%)				<0.001
Mexican American	4539(15.35)	4158 (15.41)	381 (14.82)	
Other Hispanic	2687(9.09)	2375 (8.80)	312(12.14)	
Non-Hispanic White	13238(44.78)	12105 (44.85)	1133 (44.07)	
Non-Hispanic Black	6037(20.42)	5483 (20.31)	554 (21.55)	
Other Races	3060(10.36)	2869 (10.63)	191 (7.43)	
Education Level (%)				<0.001
Less Than 9th Grade	2769(9.38)	2426 (8.99)	343(13.34)	
9–12th Grade	4089(13.83)	3560 (13.19)	529 (20.58)	
High School Grad/GED or Equivalent	6816(23.06)	6185 (22.92)	631 (24.54)	
Some College or AA degree	8922(30.18)	8132 (30.13)	790 (30.73)	
College Graduate or above	6952(23.55)	6675 (24.77)	277 (10.81)	
Marital Status (%)				<0.001
Married	15414(52.14)	14501 (53.73)	913 (35.51)	
Widowed	2240(7.58)	2011 (7.45)	229 (8.91)	
Divorced	3302(11.17)	2835 (10.50)	467 (18.16)	
Separated	968(3.27)	793 (2.94)	175 (6.81)	
Never married	5204(17.60)	4678 (17.33)	526(20.46)	
Living with partner	2425(8.20)	2164 (8.02)	261(10.15)	
Family PIR (%)				<0.001
Low (≤1.3)	9466(32.02)	8076 (29.92)	1390 (54.06)	
Medium (1.3–3.5)	10849(3670)	10031 (37.17)	818 (31.82)	
High (>3.5)	9246(31.28)	8883 (32.91)	363 (14.12)	
Smoking status (%)				<0.001
Yes	13419(45.39)	11874 (43.99)	1545 (60.09)	
No	16142(54.61)	15116 (56.00)	1026 (39.91)	
Alcohol consumption(%)				<0.001
Yes	18030(60.99)	15976(59.19)	2054(79.89)	
No	11531(39.01)	11014(40.81)	517(20.11)	
Sleep disorder (%)				<0.001
Yes	7658(25.91)	6158 (22.82)	1500(58.34)	
No	21903(74.09)	20832 (77.18)	1071(41.66)	
Hypertension (%)				<0.001
Yes	10569(35.75)	9366 (34.70)	1203 (46.79)	
No	18951(64.25)	17589 (65.17)	1362 (52.98)	
Coronary heart disease (%)				<0.001
Yes	1182(4.00)	1035 (3.83)	157 (6.11)	
No	28369(96.00)	25955 (96.17)	2414 (93.89)	
Diabetes (%)				<0.001
Yes	3751(12.69)	3251 (12.05)	500 (19.45)	
No	25810(87.31)	23739 (87.95)	2071 (80.55)	
Stroke (%)				<0.001
Yes	1065(3.60)	870 (3.22)	195 (7.58)	
No	28496(96.40)	26120 (96.78)	2376 (92.42)	
BMI(kg/m2),Mean (SD)	29.36 ± 6.98	29.12 ± 6.83	31.09 ± 8.33	<0.001
TC(mg/dL)),Mean (SD)	193.57 ± 41.99	193.32 ± 41.84	195.18 ± 44.89	<0.033
NHHR, Mean (SD)	3.12± 1.58	2.92 ± 1.43	3.12 ± 1.64	<0.001

PIR, household income to poverty ratio; BMI, body mass index; TC, total cholesterol; NHHR, nonhigh-density.

lipoprotein to HDL ratio; SD, standard deviation.

### 3.2 Association between NHHR and depression

**Figure 2:**
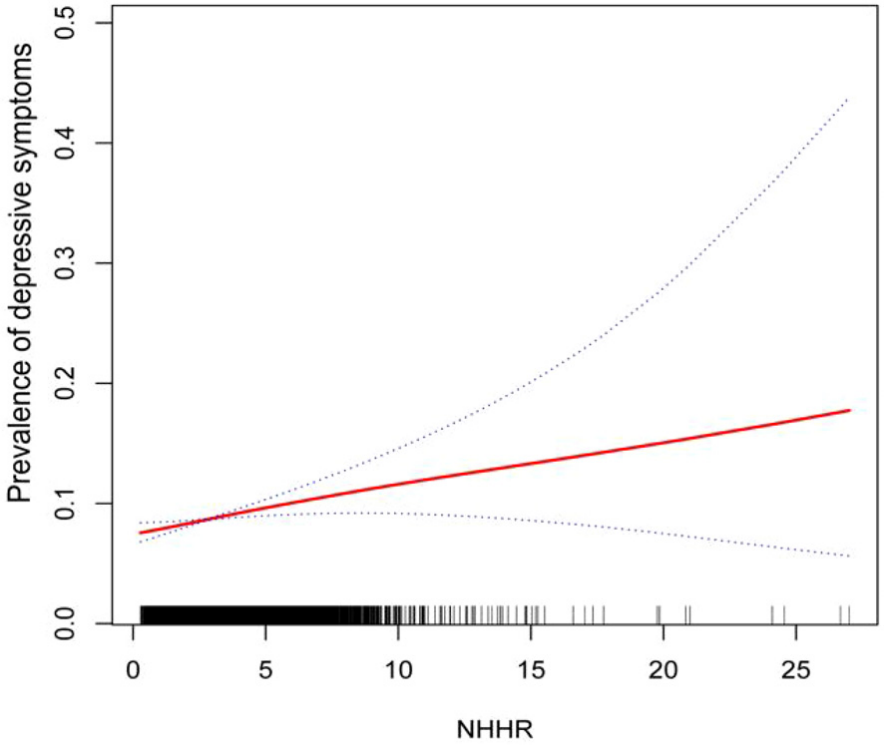
Smoothing curve fitting of NHHR index and depressive symptoms.

Our results point to a strong correlation between the prevalence of depression and NHHR. In all three models, depression has a favorable correlation with the ratio of ratios (OR) of NHHR (Model 0: OR=1.09; 95% CI (1.06, 1.12),P<0.0001; Model 1: OR=1.14; 95% CI (1.11, 1.17),P<0.0001; Model 2:OR=1.05; 95% CI (1.01, 1.09),P=0.0057). As can be seen in Model2 (fully adjusted model), the association remained statistically significant after quartiling the NHHR, with a 23% increase in the likelihood of subjects developing depression for each unit increase in NHHR through the highest quartile of NHHR compared to the lowest quartile of NHHR (**Table 2**). In addition, we further demonstrated a favorable correlation between NHHR and the prevalence of depression using smoothed curve fitting (**Figure 2**).

**Table 2:** Association between non-high-density lipoprotein cholesterol to high-density lipoprotein cholesterol ratio and depressive symptoms in different models.

Exposure	Model 0 OR (95% CI)	P value	Model 1 OR (95% CI)	P value	Model 2 OR (95% CI)	*P* value
NHHR	1.09 (1.06, 1.12)	<0.0001	1.14(1.11,1.17)	<0.0001	1.05 (1.01, 1.09)	0.0057
NHHR quartile						
Q1(0.28-1.93)	1(Ref)		1(Ref)		1(Ref)	
Q2(1.93-2.66)	1.05 (0.93, 1.18)	0.4656	1.10(0.97,1.24)	0.1253	1.01 (0.89, 1.16)	0.8391
Q3(2.67-3.65)	1.25 (1.11, 1.41)	0.0002	1.41(1.25,1.59)	<0.0001	1.20 (1.05, 1.37)	0.0072
Q4(3.65-27)	1.36 (1.22, 1.53)	<0.0001	1.67(1.48,1.88)	<0.0001	1.23 (1.06, 1.43)	0.0067
*P* for trend		<0.0001		<0.0001		0.0018

Model 0: Not adjusted.

Model 1: Adjusted for age, gender, and race.

Model 2: Further adjusted for education level, marital status, PIR, smoking status, and Alcohol consumption, BMI, Sleep disorder, Hypertension, Coronary heart disease, Diabetes, Stroke, and TC based on Model 1.

BMI, Body mass index; PIR, poverty income ratio; NHHR, non-high-density lipoprotein cholesterol to high-density lipoprotein cholesterol ratio; TC, total cholesterol; OR, Odds ratio; CI, Confidence interval.

### 3.3 Subgroup analysis

**Figure 3:**
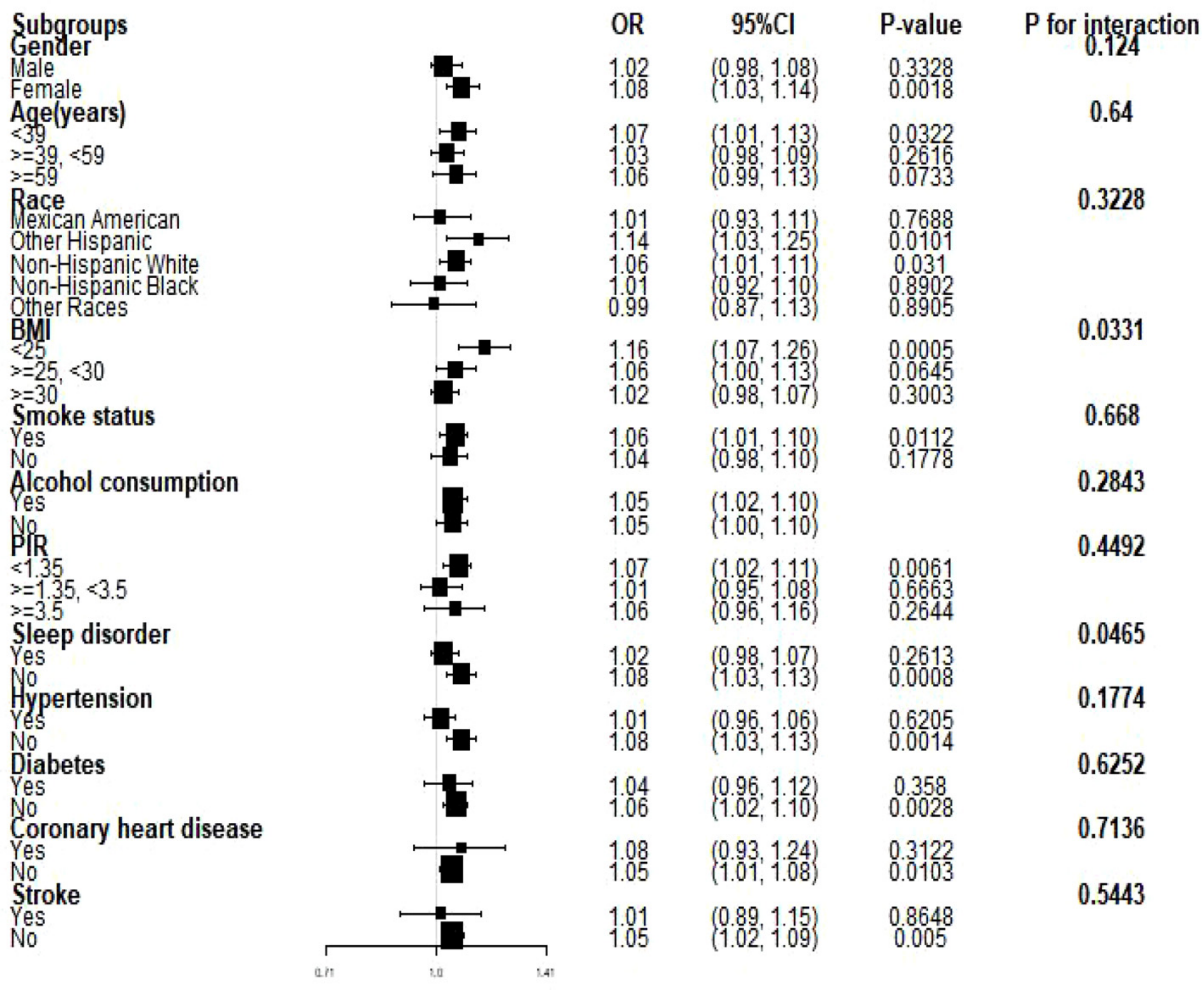
Subgroup analyses of the relationship between NHHR and the prevalence of depression. PIR, ratio of household income to poverty; BMI, body mass index. Each stratum was adjusted for all factors (age, sex, race, PIR, BMI, smoking, alcohol use, hypertension, coronary heart disease, diabetes mellitus, and stroke) except for the stratification factor itself in the model.

**Figure 4:**
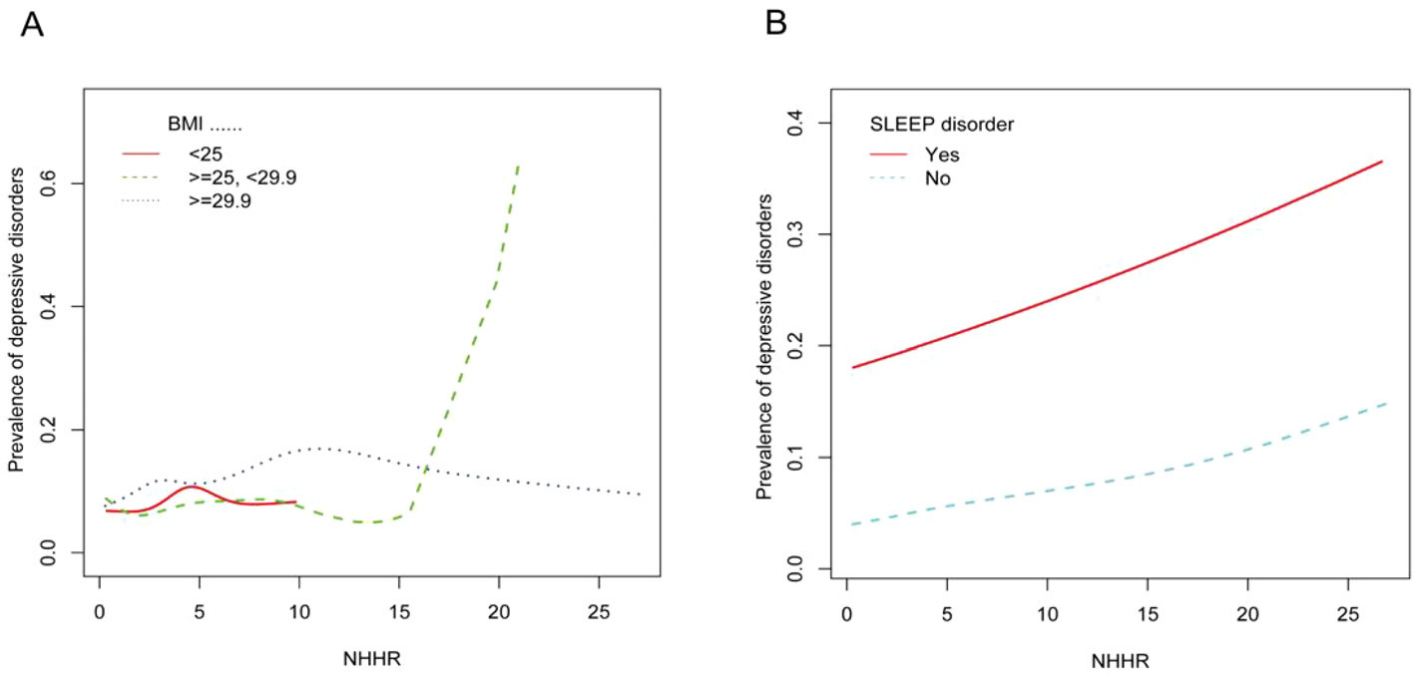
**(A)** Smoothed curves of the relationship between NHHR and the prevalence of depression analyzed stratified by BMI. **(B)** Smoothed curves of the relationship between NHHR and the prevalence of depression analyzed stratified by whether or not the subject had a sleep disorder.

We conducted subgroup analyses to assess the stability of the relationship between NHHR and the prevalence of depression across a variety of factors, including personality, age, ethnicity, BMI, smoking and drinking status, sleep disorders, coronary heart disease, hypertension, diabetes, and stroke. We found that BMI and sleep disorders altered the positive association between NHHR and the prevalence of depression (P < 0.05) (**Figures 3**, **4**).

## 4 Discussion

In our cross-sectional study of 29,561 adult participants, we found that the likelihood of participants developing depression increased as the NHHR index rose. A linear relationship between NHHR index and depression was demonstrated using smooth curve fitting. In the subgroup analyses and interaction tests conducted, we hypothesized that two stratification factors, BMI <25 kg/m2 and absence of sleep disorders, may influence the relationship between NHHR and the prevalence of depression.

This is the only study that we are aware of that evaluates the relationship between NHHR and higher rates of depression. Previous studies have discussed associations between depression and other biomarkers. The association between relative adiposity, an indicator of obesity, and depression was found to be most significant in the US adult population in a cross-sectional study conducted by Xianlin Zhu et al. (23). Depression has also been reported to be associated with lymphocyte to HDL ratios, leading to the suggestion that immune dysfunction or inflammatory factors may be a key contributor to depression (24). NHHR, as an emerging biomarker, has received much attention for its regulation of diseases such as atherosclerosis, diabetes, kidney stones, and periodontitis. Despite the lack of previous studies describing the relationship between NHHR and psychiatric disorders, there have been many reports of NHHR-related markers, including non-HDL, TC, HDL, apolipoproteins, and low-density lipoproteins. Non-HDL cholesterol is the sum of all kinds of lipoprotein cholesterol in the blood except HDL cholesterol, and relevant research investigations have found that apolipoprotein (b) and HDL, the main components of non-HDL cholesterol, are the main lipid metabolism indexes constituting atherosclerosis, and that atherosclerosis is prone to cause endothelial function damage at the beginning, and increase in vascular oxidative stress, which further causes nitric oxide-dependent diminished vasodilatory function, which in turn causes major depressive disorder (25–28). Numerous investigations have revealed that lower levels of total cholesterol, LDL and non-HDL increase the risk of depression (15, 29–31). It has been suggested that low plasma cholesterol levels can reduce the synthesis of neuromodulators such as steroids, which can lead to depressed mood (32, 33). In a cross-sectional study of the relationship between lipids and depressive episodes in postmenopausal women, it was found that after quintiles of HDL-C and LDL-C were used as continuous variables, it was concluded that lower concentrations of HDL-C and LDL-C were associated with the risk of developing depression (34). Xianlin Zhu et al. analyzed the positive association between non-HDL-C and depression in 42,143 subjects in a cross-sectional study, and the relationship was more significant in men (35). In view of the above relationship between each of the lipid indicators of depression, we believe that it is necessary for the study of the association between NHHR and depression. Specifically, the results of our subgroup analyses showed that age, gender, race, smoking, alcohol consumption, PIR, coronary heart disease, hypertension, diabetes mellitus, and stroke had no dependence on the positive correlation between NHHR and depression (P for interaction > 0.05), and the baseline characterization of participants as well as the smoothed curve fitting showed that, compared with the no depression group, subjects with depression subjects had a more significant association with NHHR and that for each unit increase in NHHR, subjects were 23% more likely to have depression. Neurosteroids refer to a class of endogenous steroids synthesized from cholesterol in the brain and nervous system. They are modulators involved In emotion, cognition, etc (36–38). Ramirez et al. found that the concentration of the neurosteroid pregnenolone in the hypothalamus was negatively correlated with BMI in animal models. That is, when BMI levels are low, they promote neurosteroid production and induce psychiatric disorders (39). Interestingly, BMI level was associated with changes in brain structure, with an inverse correlation with nucleus accumbens and insula volume in obese patients (BMI > 29.9kg/m^2^) compared with normal or underweight individuals (40, 41). The insula has been shown to play an important role in depression and cognitive function. Lidia Łapińska et al. found a positive association with insula volume in women with BMI<25 kg/m^2^. However, it was negatively correlated with Beck Depression Inventory (BDI) score (42). This was consistent with our observation that there was a positive association between NHHR and depression in people with BMI<25 kg/m^2^ (OR=1.16, 95%CI: 1.07-1.26, P < 0.01).

## 5 Advantages and limitations

Our study has a number of strengths; firstly, our data came from having a large, accurate and reliable NHANES database, and secondly, we adjusted for multiple confounding variables and performed smoothed curve fitting and subgroup analyses to validate the stability of our results and to more accurately analyze the effects of different single factors on the association between NHHR and depression. However, this paper also has limitations. First, because this paper is a cross-sectional study design, It is unable to elucidate the causal connection between NHHR and depression, and longitudinal studies with larger sample sizes are needed in the future. Second, even though we took some probable covariates into account, we are unable to totally rule out the impact of additional potential confounders. Finally, in the future, we need to further analyze the mechanism behind this correlation through experiments.

## 6 Conclusion

As a result, our initial research suggests that NHHR could be a reliable independent indicator of the risk of developing depression. Large sample sizes and more prospective research are still required to confirm our findings in the future.

## Data Availability

The datasets presented in this study can be found in online repositories. The names of the repository/repositories and accession number(s) can be found in the article/supplementary material.
